# Effect of hypoxia on integrin-mediated adhesion of endothelial progenitor cells

**DOI:** 10.1111/j.1582-4934.2012.01553.x

**Published:** 2012-09-26

**Authors:** Ralf Kaiser, Denise Friedrich, Emmanouil Chavakis, Michael Böhm, Erik B Friedrich

**Affiliations:** a Klinik für Innere Medizin V (Pneumologie), Universitätsklinikum des SaarlandesHomburg/Saar, Germany; b Klinik für Innere Medizin III (Kardiologie, Angiologie, Internistische Intensivmedizin), Universitätsklinikum des SaarlandesHomburg/Saar, Germany; c Molecular Cardiology Department of Medicine III, University of FrankfurtFrankfurt, Germany

**Keywords:** integrin, adhesion, hypoxia, EPC, AMPK

## Abstract

Homing of endothelial progenitor cells (EPCs) is crucial for neoangiogenesis, which might be negatively affected by hypoxia. We investigated the influence of hypoxia on fibronectin binding integrins for migration and cell-matrix-adhesion. AMP-activated kinase (AMPK) and integrin-linked kinase (ILK) were examined as possible effectors of hypoxia.Human EPCs were expanded on fibronectin (FN) and integrin expression was profiled by flow cytometry. Cell-matrix-adhesion- and migration-assays on FN were performed to examine the influence of hypoxia and AMPK-activation. Regulation of AMPK and ILK was shown by Western blot analysis. We demonstrate the presence of integrin β_1_, β_2_ and α_5_ on EPCs. Adhesion to FN is reduced by blocking β_1_ and α_5_ (49% and 2% of control, *P* < 0.05) whereas α_4_-blockade has no effect. Corresponding effects were shown for migration. Hypoxia and AMPK-activation decrease adhesion on FN. Although total AMPK-expression remains unchanged, phospho-AMPK increases eightfold.The EPCs require α_5_ for adhesion on FN. Hypoxia and AMPK-activation decrease adhesion. As α_5_ is the major adhesive factor for EPCs on FN, this suggests a link between AMPK and α_5_-integrins. We found novel evidence for a connection between hypoxia, AMPK-activity and integrin activity. This might affect the fate of EPCs in ischaemic tissue.

## Introduction

The increasing evidence of postnatal neovascularization leads to new therapeutic concepts to restore function of damaged organs by infusion of *ex vivo* expanded circulating endothelial progenitor cells (EPC) [1]. Human EPCs are characterized by expression of endothelium specific proteins and by their ability to react similarly to endothelial cells [2]. They have been shown to home preferentially to areas of ischaemia and to increase vasculogenesis [3]. The cells exit the blood flow and migrate through the vessel wall by a complex series of adhesion processes to vascular endothelium and extracellular matrix [4].

Firm adhesion of EPCs to the vascular endothelium and the following invasion of the vessel wall is mediated by β_2_-integrins, while rolling and light adhesion are mainly mediated by selectins. The role of β_1_-integrins in this process remains unclear [4, 5].

Nevertheless, the *ex vivo* expansion of EPCs on fibronectin (FN) as described by Asahara *et al*. requires the presence of β_1_-integrins with the subunits α_4_ and α_5_ mediating adhesion to fibronectin [2]. Furthermore, expression of β_1_ as well as α_4_ and α_5_ integrin subunits has been demonstrated on RNA level in EPCs [4]. During the process of neovascularization EPCs specifically target hypoxic tissues. This is also known from leucocytes, which enter hypoxic areas as a result of inflammatory response and its consecutive high metabolism. The efficacy of EPCs to induce neovascularization is increased after hypoxic preconditioning. This might be due to superior homing capability after accumulation of β_2_-integrins on the cell surface [6].

The AMP-activated kinase (AMPK) plays a key role in energy metabolism of cells under hypoxic conditions. Its activation results in a metabolic switch from cellular energy storage to energy release under conditions of limited ATP-supply. Hypoxia as low as 0.3–5% oxygen does not decrease bioenergetics or induce cell death, but results in mitochondrial release of reactive oxygen species. This activates LKB1 and finally AMPK by phosphorylation at Thr172 of the α-subunit [7].

In this study, the role of FN-specific integrins for adhesion and migration of EPCs as well as the influence of hypoxia was examined. AMPK and ILK were studied as possible effectors of hypoxia. The findings of this study might clarify the behaviour of EPCs in ischaemic tissue.

## Materials and methods

### Cell culture

By using the identical protocol for *in vitro* expansion of EPCs as Assmus *et al*. [8], peripheral blood mononuclear cells (MNC) from healthy human volunteers were isolated by density gradient centrifugation (1.014g/ml, Ficoll, Biocoll; Biochrom AG, Berlin, Germany), followed by three washing steps with PBS. The cells were plated at 8Mio per 75 square centimetres of fibronectin coated cell culture flasks (10 μg/ml; Sigma-Aldrich Chemie GmbH, Munich, Germany) and maintained in high protein content endothelial basal medium (Cambrex) supplemented with hydrocortisone (1 μg/ml), bovine brain extract (12 μg/ml), gentamycin (50 μg/ml), epidermal growth factor (10 ηg/ml) and 20% foetal calve serum. After 3 days, non-viable cells were removed and fresh medium added for another day before experiments were started. EPCs were characterized by double staining with FITC-labelled lectin and incorporation of 1,1′-dioctadecyl-3,3′,3′-tetramethylindocarbocyanine-labelled acetyl-low-density lipoprotein (diI-Ac-LDL). Adherent cells were incubated with DiL-Ac-LDL (2.4 ng/ml) for 1 hr and stained using FITC-labelled U. europaeus agglutinin I (lectin, 10 ng/ml) for 3 hrs.

**Fig 1:**
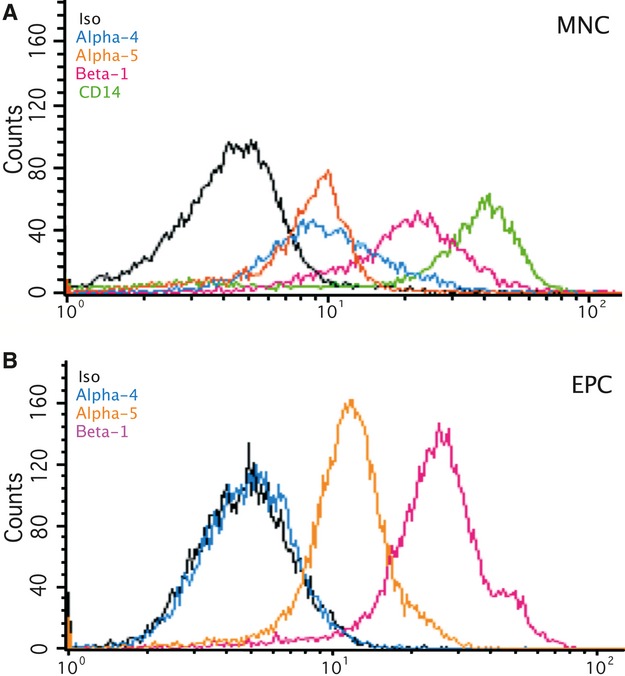
Human MNCs and *in vitro* expanded EPCs were stained with conjugated antibodies against β_1_, α_5_ and α_4_ integrins before integrin surface expression was analysed by flow cytometry. Significant lower levels of α_4_ (CD49d) were detected in EPCs. A representative FACS blot is shown.

**Fig 2:**
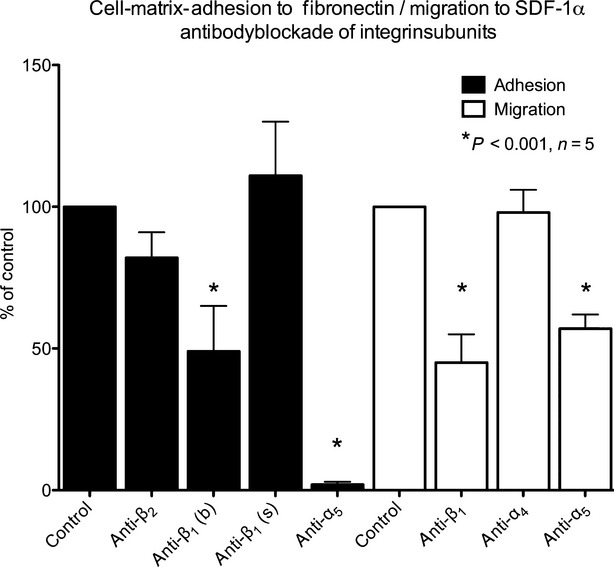
Human EPCs were pre-incubated with the indicated blocking antibodies. While blocking anti-β_1_ (CD29b) and anti-α_5_ (CD49e) decreased adhesion to fibronectin, inhibition of α_4_ (CD49d) and β_2_ (CD18) had no significant effect. A stimulating β_1_ antibody (CD29s) moderately increased adhesion (black bars). White bars show VEGF stimulated EPC-migration on fibronectin. The results parallel adhesion experiments (*n* = 5, data presented as percent of control, mean±S.D., *P < 0.05 *versus* control).

Additionally, flow cytometry showed expression of CD31, CD34 and CD18 as shown in supplementary figures (online supplement, Figs 1 and 2).

### Flow cytometry

The 3 × 10^5^ human EPCs, MNCs or cultivated monocytes were pre-incubated for 5min. with 5 μl Fc-block, washed in PBS containing 0.05% BSA and incubated for 30 min. at 4°C with 5 μl FITC-, PE- or APC-labelled antibodies (anti-β_2_, -β_1_, -α_4_, -α_5_, -CD14; BD Biosciences, Heidelberg, Germany) per 3 × 10^5^ cells. Fluorochrome labelled isotype IgG_1_ served as control. 2 ml PBS was added and cells were centrifuged for 15 min. at 4°C with 800 ×g. Cells were then resuspended in PBS containing 0.05% BSA. Flow cytometry was performed before and after 4 days of cell-type specific culture.

### Cell-matrix adhesion

The 96-well plates (Costar Corning, Amsterdam, The Netherlands) were coated overnight at 4°C with 10 μg/ml recombinant fibronectin (Sigma-Aldrich) in phosphate buffered saline (PBS). Wells were washed with PBS once and 50 μl adhesion buffer was added to prevent drying. *Ex vivo* expanded EPCs were stained with 5 μl CellTracker™ green (CMFDA; Molecular Probes/Invitrogen, Life Technologies, Darmstadt, Germany) per 3 ml culture medium for 5 min. and detached with trypsin. Trypsin was blocked, cells washed and resuspended in adhesion buffer (150 mM NaCl, 20 mM Hepes, 2 mM MgCl_2_, 0.05% fraction V bovine serum, 5% Glucose, pH 7.42). Experiments were performed as indicated in triplets with 10^5^ cells per well. For inhibition experiments, EPCs were pre-incubated with antibodies for 15 min. at 4°C. Anti-α_5_ (CBL497, clone SAM-1), anti-β_2_ (CBL158, clone MEM-48), anti-β_1_ (CBL481, clone TDM29), anti-α_4_ (MAB1954Z, clone P4G9), anti-α_5_ (MAB1956Z, clone P1D6), stimulating anti-β_1_ (MAB1951Z, clone P4G11), blocking anti-β_1_ (MAB2253Z, clone 6S6) were obtained from Chemicon International. Goat IgG (R&D Systems, Minneapolis, MN, USA) served as control. Alternatively, experiments with cyclic RGD peptide (Sigma-Aldrich) at 50 μM were performed. Pre-incubation was performed similar to antibody treatment. After pre-incubation, the plate was centrifuged for 3 min. at 300 r.p.m. (RZF 17 g) to achieve simultaneous contact of the cells to the plate. After 20 min. of incubation at 37°C, non-adherent cells were removed and the plates were washed twice with adhesion buffer and fixed with 4% paraformaldehyde. Adherent cells were quantified by fluorescence microscopy (magnification 20×, Axiovert 100; Carl Zeiss, Oberkochen, Germany). Cells were counted in five randomly selected fields covering different areas of each well. Images were digitized and the software package ScionPro with custom macros was used for semi-automated counting. Data were normalized and presented as percentage of control.

### Cell migration assay

The assays were performed as previously described [4]. Transwell membranes (8 μm; Costar) were coated with fibronectin (10 μg/ml; Sigma-Aldrich, Germany) overnight at 4°C. *Ex vivo*– expanded human EPCs were stained with 5 μl CellTracker™ green (5-chloromethylfluorescein diacetate, Molecular Probes, Invitrogen) per 3 ml culture medium for 5 min. at 37°C. EPCs were detached by trypsinization. Trypsin was neutralized and 1.2x10^5^ EPCs were resuspended in 100 μl serum-free RPMI 1640 containing 0.05% BSA. Serum-free RPMI 1640 (600 μl) with 0.05% BSA containing 50 ng/ml of SDF-1α was placed in the lower chambers.

Cells were incubated at 37°C in 5% CO_2_ for 12 hrs. For inhibition experiments, EPCs were pre-incubated for 15 min. at 4°C with 5 μl of the indicated antibodies per 3.0 × 10^5^ cells. The antibodies were free of sodium azide to reduce toxicity during the assay. Cells remaining on the upper surface of the filters were mechanically removed, and migrated cells at the lower surface were fixed with 4% formaldehyde and counted in five fields by using a fluorescence microscope (Axiovert 100; Carl Zeiss).

### Hypoxic conditioning

According to the requirements of the experiment either adherent (Western blots) or resuspended cells (cell-matrix-adhesion, migration) were incubated in hypoxic environment. An electronically regulated incubator was used (Model C42; Labotect, Göttingen, Germany) to determine temperature and concentration of oxygen and carbondioxide during the experiments. Controls were preserved in regular incubators (37°C, 5% carbondioxide, oxygen as indicated, nitrogen ad 100%). The incubator was calibrated prior to each experiment.

### Statistical analysis

Data were calculated using Microsoft™ Excel for Mac and statistical calculations were performed by Graphpad™ Prism (GraphPad Software Inc, La Jolla, CA, USA). Data are presented as mean ± S.D. Comparisons between groups were calculated using ANOVA with Bonferroni correction for multiple testing. For non-parametric distributions tests were used as appropriate.

## Results

### Integrin profile of EPCs

As revealed by flow cytometry, EPCs express β_1_ and α_5_ but not α_4_ (Fig. 1). In contrast, MNCs before and after non-cell-specific cultivation show unaltered expression of both α_4_ and α_5_. Both cell types were positive for β_2_ (see online supplement). Preselected cells expressing CD14 showed a similar expression of integrin subunits (Suppl. Fig. 6).

### Functionality of expressed integrins

The EPCs were pretreated with integrin specific antibodies and data were normalized for control. As expected, there is no significant loss of adhesion to fibronectin due to blockade of β_2_ (81.8 ± 9.3% of control). Inhibition of β_1_ leads to partial loss of adhesion and inhibition of α_5_ to a complete ablation of adhesive capacity to fibronectin (49.4 ± 15.8% and 2.1 ± 1.3% respectively). The use of different clones of antibodies leads to the same results (clone TDM29 and clone 6S6 for β_1_, clone SAM-1 and clone P1D6 for α_5_), although application of a stimulating antibody targeting β_1_ leads to a slight albeit statistically not significant increase of adhesion (111.2 ± 18.7%). α_4_-blockade does not alter adhesion (106 ± 26%). In consistency with the expression profile seen in flow cytometry, only inhibition of the expressed fibronectin-specific integrins β_1_ and α_5_ leads to a significant loss of adhesion in this model. The effect of α_5_-blockade on EPCs was much higher than blockade of β_1_. In contrast, blockade of each of the integrin subunits α_4_, α_5_ and β_1_ showed to diminish adhesion to fibronectin in peripheral blood MNC. Using a cyclic RGD peptide, adhesion to fibronectin decreased by 50% in endothelial progenitor cells (see online supplement, Figs 4 and 5).

To determine the role of the expressed integrins in the dynamic process of migration, EPCs migrated in a fibronectin coated modified Boyden chamber along a SDF-α-gradient. Migration is markedly reduced when β_1_ or α_5_ are blocked (45.4 ± 22.1% and 57.5 ± 9.1% of control, respectively). Blockade of α_4_ showed no effect on migration capacity (98.4 ± 16.9% of control, Fig. 2).

### Adhesion of EPCs under hypoxic conditions

**Fig 3:**
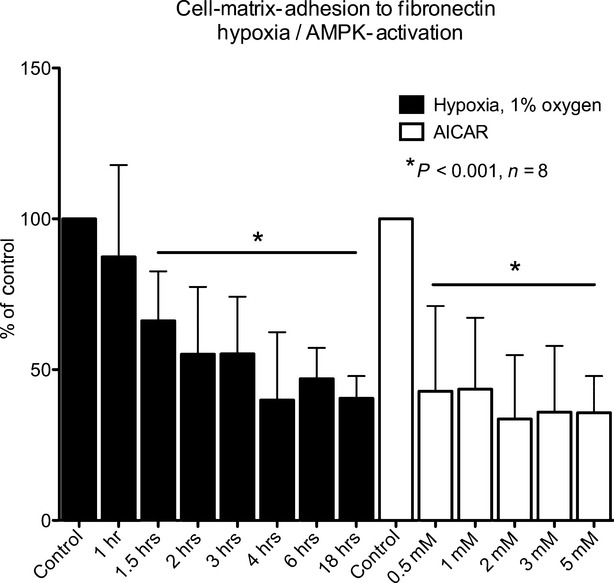
Human EPCs were either submitted to hypoxia (black) or stimulated with AICAR (white). Integrin activity was tested by adhesion to fibronectin. Increase of AMPK-activity by both hypoxia and AICAR-stimulation decreases integrin activity (*n* = 8, data presented as percent of control, mean ± S.D., *P < 0.05 *versus* control).

We further investigated the effects of hypoxia on integrin activity. *Ex vivo* expanded human EPCs were subjected to hypoxia. The static adhesion assay was performed as described above. With increasing duration of hypoxia, EPCs are less able to adhere to fibronectin in this experiment. Statistical significance is reached at 1.5 hrs of 1% oxygen (Fig. 3). After 2 hrs, adhesion capacity is reduced to 55.1 ± 22.3% of control and reaches a minimum of 39.9 ± 22.5% of control after 4hrs. Further increase of time under hypoxia up to 12 hrs had no additional effect. The cells show no signs of necrosis or apoptosis as determined by Roche® Cell death detection kit (see online supplement, Fig. 3).

As the onset of this effect is too early to be caused by altered protein translation, we focused on protein interactions. AMP-dependent kinase is a sensor molecule for intracellular energy content expressed as ADP/ATP and AMP/ATP ratio. It is stimulated by hypoxia and the highly specific activator aminoimidazole carboxamide ribonucleotide (AICAR). *In vitro* expanded EPCs were treated with increasing doses of AICAR for 4hrs and then tested for their adhesion capacity to fibronectin. Even low doses of AICAR result in diminished integrin activity beginning with 42.8 ± 23.7% at 0.5 mM AICAR to 35.7 ± 12.2% at 5 mM AICAR (Fig. 3).

### Activation of AMP in EPCs under hypoxic conditions

**Fig 4:**
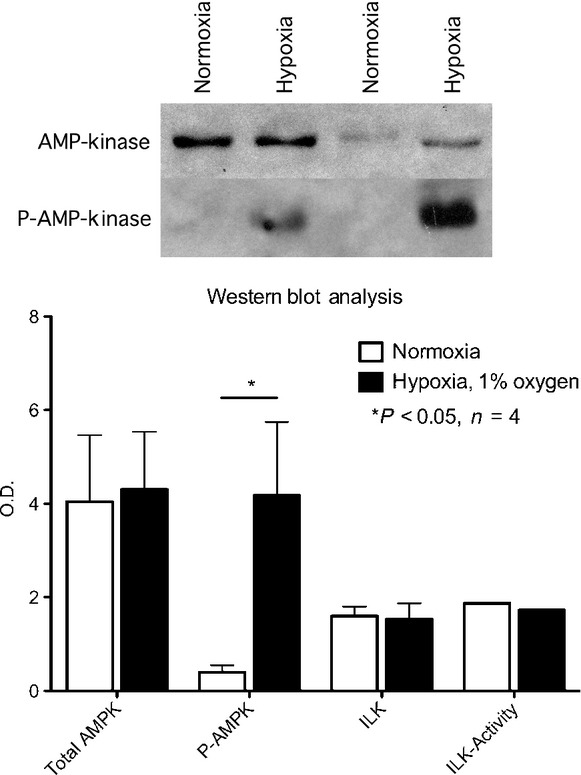
Western blot analyses showed unaltered total level of AMPK but an increase of phosphorylation at an activating phosphorylation site. ILK-activity as shown by GSK3-assay remained unchanged.

Activation of AMPK by phosphorylation at Thr172 has been shown in other cell types to activate AMPK during hypoxia. In EPCs the level of phosphorylation increased eightfold, whereas total expression remained unchanged (Fig. 4). As demonstrated by GSK3-assay, ILK-activity was not altered by hypoxia (Fig. 4).

## Discussion

In this study we were able to demonstrate the expression of fibronectin-specific integrins on EPCs and the markedly decreased expression of α_4_ compared to MNCs. We further showed the functional relevance of this finding for adhesion and migration on FN. Both hypoxic and pharmacological AMPK-activation lead to diminished activity of these integrins. We finally demonstrated the phosphorylation and therefore activation of AMPK during hypoxia. Our results add to present knowledge about behaviour of EPCs under hypoxic conditions as found in targeted tissues for cell based therapy.

In this study we were able to verify the expression of α_5_- and β_1_-integrins by flow cytometry. Although MNCs also expressed α_4_-integrin, this could not be seen after endothelium specific culture of the cells. Despite the previously described heterogeneity of *ex vivo* expanded early EPCs [9], our finding represents a common denominator of these subpopulations similar to their endothelial surface markers. Chavakis *et al*. compared the mRNA expression of EPCs to HUVECs by using a microarray. In that investigation, both β_2_ and CD11b/c were significantly increased in EPCs compared to HUVECs on mRNA level, whereas β_1_ and α_5_ were slightly decreased without statistical significance. α_4_-integrin was slightly increased without reaching statistical significance.

We perceived a high expression of β_1_ and α_5_, whereas α_4_ was markedly decreased during expansion. Both α_4_β_1_ and α_5_β_1_ integrins have a high affinity to fibronectin [10], which is an important regulator of various cellular processes including survival, differentiation, growth and migration. It is deposited actively in the ECM by cells and circulates freely in the plasma. Recent data suggested an important role in flow-induced vascular remodelling by influencing the invasion of leucocytes and the proliferation of vascular smooth muscle cells [11]. Most importantly, it is used as immobilized form for the *in vitro* expansion of EPCs [8]. Therefore, we investigated the functional relevance of the altered integrin expression. Our findings emphasize the importance of α_5_β_1_ integrins for EPCs to adhere on the provided fibronectin matrix during *in vitro* expansion. Interestingly, inhibition of α_5_ shows a markedly pronounced effect on the cells compared to blockade of β_1_. Furthermore, the possibility of an insufficient affinity of the antibody was addressed by comparing different clones of blocking antibodies without any effect on this finding. Hence, one can speculate about the signalling properties of integrins and the effects on the signalling pathways of such blockage. These pathways have been subject to investigation in case of β_1_, but data on α_5_ remain scarce [12, 13]. The used cell culture protocol has been shown to produce subpopulations of EPCs probably because of the short time for differentiation. The phenotypic characterization with both functional assays (diLDL-uptake) and flowcytometry for endothelial surface markers showed a high content of endothelial type cells (see online supplement) in concordance with previous literature [9]. The integrin profiles were found irrespective of this heterogeneity, but further analysis of subpopulations may help our understanding of cell based tissue regeneration.

Migration comprises a series of complex actions of the cell. It depends on a coordinated sequence of adhesion and release of molecules on the cell surface as well as the cell moves along a chemotactic gradient. This process, in which integrins and selectins are important effectors, is only incompletely understood [14]. In our experiments with EPCs we demonstrated the functional relevance of β_1_-integrins for migration on a fibronectin matrix. The inhibition of both β_1_ and α_5_ but not α_4_ resulted in decreased adhesion and migration capacity of EPCs. This finding matches the expression profile.

Akita and coworkers found an increased efficacy of EPCs regarding vasculogenesis after hypoxic preconditioning. This was mainly due to an accumulation of β_2_-integrins [6]. Kong *et al*. investigated the effects of hypoxia on the integrin expression of leucocytes. They reported an up-regulation of β_2_ but not β_1_ under hypoxic conditions. Additionally they found an increased β_2_-integrin dependent increase of adhesion to endothelial cells after hypoxia [15]. Taking into account the importance of β_2_-integrins for the homing of EPCs [4], this mechanism explains the effect of hypoxic preconditioning of EPCs. The effect of hypoxia on β_1_-integrins remained unclear. In this study, we subjected EPCs to hypoxic conditions and demonstrated a decreased adhesion of EPCs on fibronectin. As the adhesion to fibronectin of these cells was strictly dependent from α_5_β_1_ integrins, we deducted an influence of hypoxia on expression or function of these integrins. Consistent with the aforementioned literature, no increase of β_1_ or α_5_ could be detected after hypoxia (Suppl. Fig. 7A–C).

The rapid onset of the effect leads to the hypothesis of protein modifications due to hypoxia. Hypoxia activates AMP-dependent kinase (AMPK) in EPCs, which is a sensor molecule for metabolic stress and energy level [7]. Recent data suggest a role of mitochondrial ROS-release during hypoxia as the main activator of AMPK in this setting [7]. Hypoxia leads to phosphorylation of AMPKα at Thr172 and enhances its enzymatic activity. This influences an extensive number of pathways [16–19]. Aminoimidazole carboxamide ribonucleotide (AICAR) is a specific activator of AMPK. In our experiments we demonstrated the same dose-dependent reduction of adhesion by AICAR as was seen in hypoxia. As described before, we observed phosphorylation of AMPK on Thr172 during hypoxia. Taking into account these findings, we conclude that upon activation AMPK affects cell-matrix adhesion directly or indirectly by modification of either α_5_ or β_1_.

Several phosphorylation sites on the cytosolic domains are believed to control conformation and alignment of the branches of α- and β-subunit causes altered adhesion [10]. Neither the exact impact of phosphorylation on these sites nor the phosphorylating enzymes have been sufficiently examined in this context. Our study contributes to knowledge of inside-out signalling of integrins. We have previously reported the down-regulation of the active conformation of β_1_ in response to *ex vivo* deletion of ILK in endothelial cells. The mechanism led to apoptosis of the cells and was independent of akt [20]. Our experiments show neither decreased ILK expression in response to hypoxia nor decreased ILK-activity in contrast to previous reports [21] (Fig. 4).

Increasing intensity of both hypoxia and AICAR leads to marked reduction of adhesion. But this effect did not abolish the adhesion capacity. Therefore, one might speculate whether inactivation of β_1_ or α_5_ might be the possible mechanism of hypoxia and whether activation of AMPK is causative for this.

As endothelial nitric oxide synthetase is activated by AMPK [22, 23], we carried out adhesion experiments using nitric oxide donators and found no impact on adhesion. Therefore, not only eNOS but also most heme-group dependent enzymes appeared unlikely targets.

In summary, we demonstrated the expression of α_5_β_1_-integrin on *ex vivo* expanded EPCs in contrast to α_4_β_1_ and α_5_β_1_ on MNCs. We were able to demonstrate the importance of α_5_ for adhesion to fibronectin as well as the influence of hypoxia on functional capacity of α_5_β_1_-integrin. We then confirmed the activation of AMPK by phosphorylation of AMPKα-Thr172 in response to hypoxia in EPCs. Finally, data could be presented implicating a causative relation of AMPK and function of α_5_β_1_-integrin. As previously described, α_5_β_1_-integrins might not be important for the homing of EPCs, as their adhesion to endothelium is not affected. But binding of fibronectin to α_5_β_1_-integrins might play a role in cell growth and differentiation of EPCs during tissue repair. The influence of hypoxia on α_5_β_1_-integrins and its downstream targets LKB1, mTOR, eNOS, KLF and many more remains subject of further investigation.

## Supporting information

**Figure S1** Characterization of EPCs (**A**–**D**) Representative fluorescence microscopic images showing DiI-Ac-LDL uptake and Ulex europaeus-lectin binding of adherent cells as well as typical spindle-shaped endothelial cell-like morphology after 4 days of cell culture under endothelial selection pressure (100× magnification). A: green – Ulex europaeus-lectin, B: red – DiI-Ac-LDL, C: DAPI counterstaining, D: fusion image of A, B and C.

**Figure S2** Characterization of EPCs (**A**–**C**) Representative flowcytometry analysis showing iso-IgG1 (**A**), co-staining of CD31-FITC (B&D) and CD34-APC (B&D) (**B**) and co-staining of CD31-FITC and CD18-APC (B&D) (**C**). As described previously, EPCs express highly CD31, CD34 and CD18 (beta2-integrin subunit) after *in vitro* expansion.

**Figure S3** Cell death and apoptosis. Two experiments were performed to detect cell death during hypoxia at 1% oxygen for 8 hrs. The assay was performed following vendor instructions (Roche, Roche Diagnostics Deutschland GmbH, Mannheim, Germany).

**Figure S4** Cell-matrix-adhesion to fibronectin. Peripheral blood mononuclear cells (MNC) and *in vitro* expanded endothelial progenitor cells (EPC) from the same individuals were compared. Integrin subunits were blocked selectively by antibodies by pre-incubation. Although adhesion is blocked by antibodies targeting α5 and β1, only MNCs are blocked by the anti-α4-antibody.

**Figure S5** Cell-matrix-adhesion to fibronectin. EPCs were pre-incubated with 50 μM of cyclic RGD-peptide (Sigma-Aldrich), an established inhibitor of αVβ3, αVβ5, α4β1 and α5β1. EPCs show a decreased adhesion capacity to the fibronectin matrix, when RGD-recognizing integrins are blocked.

**Figure S6** Flow cytometry for integrin subunits. Peripheral blood mononuclear cells were isolated by density gradient. The cells were enriched by CD14 labelling by using magnetic beads. After 4 days of endothelial specific culture, flow cytometry was performed (see methods). CD14-enriched *in vitro* expanded cells demonstrated the same integrin profile as endothelial progenitor cells obtained from the Asahara protocol (Fig. 1).
