# Supplementary material for: Effect of hypoxia on integrin-mediated adhesion of endothelial progenitor cells
Source: J Cell Mol Med. 2012 Sep 26;16(10):2387–93. doi: 10.1111/j.1582-4934.2012.01553.x (PMC3823432; doi:10.1111/j.1582-4934.2012.01553.x)
Supplement: Supplementary file 1 [file jcmm0016-2387-SD1.doc]

**Online Supplementary Figure Legends**

**Online Supplementary Figure 1.**

Characterization of EPCs **A-D.** Representative fluorescence microscopic images showing DiI-Ac-LDL uptake and *Ulex europaeus* lectin binding of adherent cells as well as typical spindle-shaped endothelial cell-like morphology after 4 days of cell culture under endothelial selection pressure (100x magnification).

A: green – *Ulex europaeus*-lectin, B: red – DiI-Ac-LDL, C: DAPI counterstaining, D: fusion image of A, B, and C.
