# Supplementary material for: Effect of hypoxia on integrin-mediated adhesion of endothelial progenitor cells
Source: J Cell Mol Med. 2012 Sep 26;16(10):2387–93. doi: 10.1111/j.1582-4934.2012.01553.x (PMC3823432; doi:10.1111/j.1582-4934.2012.01553.x)
Supplement: Supplementary file 2 [file jcmm0016-2387-SD2.doc]

**Online Supplementary Figure Legends**

**Online Supplementary Figure 2.**

Characterization of EPCs **A-C.** Representative flowcytometry analysis showing iso-IgG_1_ (A), costaining of CD31-FITC (B&D) and CD34-APC (B&D) (B) and co-staining of CD31-FITC and CD18-APC (B&D) (C). As described previously, EPCs express highly CD31, CD34 and CD18 (beta_2_-integrin subunit) after in vitro expansion.
