# Supplementary material for: Effect of hypoxia on integrin-mediated adhesion of endothelial progenitor cells
Source: J Cell Mol Med. 2012 Sep 26;16(10):2387–93. doi: 10.1111/j.1582-4934.2012.01553.x (PMC3823432; doi:10.1111/j.1582-4934.2012.01553.x)
Supplement: Supplementary file 3 [file jcmm0016-2387-SD3.doc]

**Online Supplementary Figure Legends**

**Online Supplementary Figure 3.**

Cell death and apoptosis. Two experiments were performed to detect cell death during hypoxia at 1% oxygen for 8 hours. The assay was performed following vendor instructions (Roche, Germany).
