# Supplementary material for: Effect of hypoxia on integrin-mediated adhesion of endothelial progenitor cells
Source: J Cell Mol Med. 2012 Sep 26;16(10):2387–93. doi: 10.1111/j.1582-4934.2012.01553.x (PMC3823432; doi:10.1111/j.1582-4934.2012.01553.x)
Supplement: Supplementary file 4 [file jcmm0016-2387-SD4.doc]

**Online Supplementary Figure Legends**

**Online Supplementary Figure 4.**

Cell-matrix-adhesion to fibronectin. Peripheral blood mononuclear cells (MNC) and in vitro expanded endothelial progenitor cells (EPC) from the same individuals were compared. Integrin subunits were blocked selectively by antibodies by pre-incubation. While adhesion is blocked by antibodies targeting α_5_ and β_1_, only MNCs are blocked by the anti-α_4_-antibody.
