# Supplementary material for: Effect of hypoxia on integrin-mediated adhesion of endothelial progenitor cells
Source: J Cell Mol Med. 2012 Sep 26;16(10):2387–93. doi: 10.1111/j.1582-4934.2012.01553.x (PMC3823432; doi:10.1111/j.1582-4934.2012.01553.x)
Supplement: Supplementary file 5 [file jcmm0016-2387-SD5.doc]

**Online Supplementary Figure Legends**

**Online Supplementary Figure 5.**

Cell-matrix-adhesion to fibronectin. EPCs were pre-incubated with 50μM of cyclic RGD-peptide (Sigma-Aldrich), an established inhibitor of αVβ_3_, αVβ_5_, α_4_β_1_ and α_5_β_1_. EPCs show a decreased adhesion capacity to the fibronectin matrix, when RGD-recognising integrins are blocked.
