# Supplementary material for: Effect of hypoxia on integrin-mediated adhesion of endothelial progenitor cells
Source: J Cell Mol Med. 2012 Sep 26;16(10):2387–93. doi: 10.1111/j.1582-4934.2012.01553.x (PMC3823432; doi:10.1111/j.1582-4934.2012.01553.x)
Supplement: Supplementary file 6 [file jcmm0016-2387-SD6.doc]

**Online Supplementary Figure Legends**

**Online Supplementary Figure 6.**

Flow cytometry for integrin subunits. Peripheral blood mononuclear cells were isolated by density gradient. The cells were enriched by CD14 labeling using magnetic beads. After 4 days of endothelial specific culture, flow cytometry was performed (see methods). CD14-enriched in vitro expanded cells demonstrated the same integrin profile as endothelial progenitor cells obtained from the Asahara protocol (Figure 1).
