# Supplementary material for: Effect of hypoxia on integrin-mediated adhesion of endothelial progenitor cells
Source: J Cell Mol Med. 2012 Sep 26;16(10):2387–93. doi: 10.1111/j.1582-4934.2012.01553.x (PMC3823432; doi:10.1111/j.1582-4934.2012.01553.x)
Supplement: Supplementary file 7 [file jcmm0016-2387-SD7.doc]

**Online Supplementary Figure Legends**

**Online Supplementary Figure 7.**

Flow cytometry for integrin subunits. Effect of hypoxia on integrin expression of EPCs. Normoxia is shown in blue, 18h hypoxie at 1% oxygen is shown in brown. No differences in surface expression of integrin subunits could be detected. **A:** IgG control, **B:** integrin β_1_-integrin subunit, **C:** α_5_-integrin subunit.
